# Consequences of Genomic DNA Mono-Ribonucleotides for Chromosomal Stability

**DOI:** 10.1093/geroni/igab046.3574

**Published:** 2021-12-17

**Authors:** Tavia Roache

**Affiliations:** Howard University, Washington, District of Columbia, United States

## Abstract

Mono-ribonucleotides are building blocks for polynucleotide RNA chains (e.g., messenger RNA), but if mis-incorporated into duplex DNA can cause mutagenesis and chromosomal instability. During DNA synthesis by Pol γ, remnants of unremoved RNA primers contribute to elevated mono-ribonucleotide triphosphates resulting in nucleotide pool imbalance, ultimately favoring mis-incorporated ribonucleotides during replication. Moreover, although polymerases generally replicate DNA with high fidelity, the steric gate occasionally allows a mis-incorporated ribonucleotide. Thus, a mono-ribonucleotide is one of the most abundant lesions in genomic DNA of eukaryotes. If unremoved from double-stranded DNA, the ribonucleotide exerts negative effects on replication, transcription, and genomic maintenance, with lasting effects on cellular homeostasis. Even a single ribonucleotide in telomeric DNA comprises shelterin binding and telomere capping causing vulnerability to spontaneous hydrolysis which potentiates telomere shortening. Consistent with this, a ribonucleotide positioned in double-helical DNA alters its structure by torsinally distorting the sugar-phosphate backbone. Fortunately, cellular response and repair pathways exist to help cells cope with mis-incorporated mono-ribonucleotides. The Ribonucleotide Excision Repair (RER) or a Topoisomerase 1 (Top1)-mediated pathway remove embedded ribonucleotides. For RER, RNase H2 incises 5’ of a mono-ribonucleotide, creating an access point for its removal. If cells are deficient in RNase H2, Top1 initiates removal of the ribonucleotide. However, Top1 is less accurate than RNase H2, which can lead to mutagenesis. Studying the mechanisms in which ribonucleotides are incorporated into DNA or further metabolized should provide insight to their negative consequences for chromosomal integrity, cancer, and auto-immune disease attributed to a genetic deficiency of RNase H2.

